# Integrated Metabolomic and Transcriptomic Analysis Reveals Differential Flavonoid Accumulation and Its Underlying Mechanism in Fruits of Distinct *Canarium album* Cultivars

**DOI:** 10.3390/foods11162527

**Published:** 2022-08-21

**Authors:** Ruilian Lai, Chaogui Shen, Xin Feng, Minxia Gao, Yongyan Zhang, Xiaoxia Wei, Yiting Chen, Chunzhen Cheng, Rujian Wu

**Affiliations:** 1 Fruit Research Institute, Fujian Academy of Agricultural Sciences, Fuzhou 350013, China; 2 College of Horticulture, Shanxi Agricultural University, Jinzhong 030801, China

**Keywords:** *Canarium album*, flavonoid, metabolomics, transcriptomics, gene expression

## Abstract

*Canarium album* fruit has great potential to be consumed as a raw material not only for food but also medicine. The diverse active metabolites composition and content of *C. album* fruits greatly affect their pharmacological effects. However, up to now, there has been no report on the global metabolome differences among fruits from distinct *C. album* cultivars. In our present study, by using non-targeted metabolomics techniques, we identified 87 DAMs (differentially accumulated metabolites) including 17 types of flavonoids from fruits of four different *C. album* cultivars. KEGG (Kyoto Encyclopedia of Genes and Genomes) pathway enrichment analysis revealed that the flavone and flavonol biosynthesis- and flavonoid biosynthesis-related DAMs were major factors determining their metabolome differences. Comparative transcriptomic analysis revealed that 15 KEGG pathways were significantly enriched by genes of the identified 3655 DEGs (differentially expressed genes) among different *C. album* cultivars. Consistent with the metabolome data, flavonoid biosynthesis-related DEGs, including eight key structural genes (such as *FLS*, *CCoAOMT*, *CHI*, *C4H*, *DFR*, *LAR*, and *C3′H*, etc.) and several regulatory transcription factor (TF) genes (including 32 *MYBs* and 34 *bHLHs*, etc.), were found to be significantly enriched (*p* < 0.01). Our study indicated that the differential expression of flavonoid biosynthesis-related genes and accumulation of flavonoids played dominant roles in the various metabolome compositions of fruits from different *C. album* cultivars.

## 1. Introduction

*Canarium album*, belonging to the Burseraceae family, is a typical tropical and subtropical fruit tree originated from China and is mainly cultivated in the Fujian, Guangdong, Guangxi, Sichuan, Chongqing, and Zhejiang provinces of China [1]. Its fruit has a thick flavor and sweet aftertaste, and is very popular in these areas. In addition to its edibility, *C. album* fruit also has good medicinal values and is recorded as an important traditional Chinese medicine material in the Compendium of Materia Medica compiled by Shizhen Li. Recent reports have also proved that *C. album* fruit contains many active ingredients. Its extract could prevent obesity development, ameliorate metabolic dysfunction in diabetes, alter the gut microbiota composition, and ameliorate the metabolic abnormalities associated with fatty liver under high-fat challenge [2,3,4,5]. Sitoindoside I, amentoflavone, tetrahydroamentoflavone, protocatechuic acid, and benzofuran neolignans in fruit of *C. album* have anti-inflammatory effects [6,7], while the polysaccharide and ethyl acetate in it could be, respectively, exploited as valuable antioxidant and antibacterial components [8,9]. Besides, some phenylpropanoids of *C. album* fruit show remarkable anti-neuroinflammatory and antidiabetic activities [10]. It can be seen that *C. album* fruit has high medicinal value because of the abundant and diverse active metabolites, which are the basis for their pharmacological effects. Currently, studies on *C. album* metabolites focus mainly on the extraction method improvement, pharmacological activity analysis of specific components, and quantitation of some known compounds, and so on. For instance, He et al. modified the microwave-assisted extraction method of polyphenols and identified the main polyphenol components of *C. album* [11]. Yang et al. obtained ten known metabolite compounds, such as ellagic acid, gallic acid, and scopolamine, from *C. album* fruits by using repeated chromatography [12]. Chen et al. isolated isocorilagin and methyl brevifolincarboxylate from *C. album* [13,14]. It should be noted that the metabolite type and content differences in fruits from different cultivars greatly influence their functional activities [15]. However, the global metabolome differences among fruits from different *C. album* cultivars and their underlying mechanism have received much less attention.

With the rapid development of molecular biology technologies, metabolomic and transcriptomic analyses have been widely used in the studies of genes that regulate the metabolite biosynthesis of plants [16]. By integrating the data generated from both techniques, it is possible to reveal the differences in metabolic components between different biological samples and to clarify the underlying molecular mechanisms [17,18,19]. The metabolomics technology is a powerful tool that can be used to explore the differences in composition and content of metabolic components in different samples, and to clarify the mechanism underlying certain phenomena, such as fruit quality formation, biotic/abiotic stress responses, variety specificity, and so on [20]. Using an integrated analysis of the transcriptome and metabolome, Zhao et al. revealed the changes in the accumulation of metabolites and the expression of metabolites biosynthesis-related genes during fruit ripening of *Lycium ruthenicum* and *L. barbarum*, and laid a foundation for the exploration of genetic color variation mechanisms [21]. Jia et al. explored the key genes regulating white petal color in *Brassica napus* and provided important insights into the molecular mechanisms of the carotenoid metabolism pathway associated with color variations in rapeseed petals [22]. Bai et al. compared the differences in *Pinus massoniana* xylem with high, medium, and low resin-yielding capacities, and proposed that PKc-, and LRR-RLK-related regulatory and metabolic pathway may play key roles in oleoresin biosynthesis [23]. Zhu et al. identified the active component differences in different *Angelica sinensis* varieties, and established the flavonoid regulation network, which provided references for flavonoid production and variety selection of *A. sinensis* [24].

Transcriptomic techniques have been successfully applied in the study of *C. album* [25,26]. However, up to now, no metabolomic research has reported the metabolome differences in different *C. album* cultivars, which has greatly limited the development of biotechnological applications for this valuable fruit crop. In the present study, to provide a scientific basis for directional breeding, germplasm innovation, and the use of *C. album*, the differential metabolic components of fruits from four main traditional cultivars, *C. album*. cv. ‘Changying’ (C), ‘Tanxiang’ (T), ‘Huiyuan’ (H), and ‘Zilaiyuan’ (Z), were analyzed and compared by using non-targeted metabolomics technology, and the molecular mechanism as well as the DEGs (differentially expressed genes) directly or indirectly regulating the biosynthesis of DAMs (differentially accumulated metabolites) were clarified through the integration of transcriptomics and metabolomics data. The results obtained in this study will be helpful for understanding the underlying mechanism of differential metabolite accumulations in different *C. album* cultivars and can provide a basis for the future highly active compounds focused on *C. album* breeding.

## 2. Materials and Methods

### 2.1. Materials

Fruits of *C. album*. cv. ‘Changying’ (C), ‘Huiyuan’ (H), ‘Tanxiang’ (T), and ‘Zilaiyuan’ (Z) were collected from Fuzhou Germplasm Repository of Chinese Olive of Agriculture Ministry of China (26°07′36.70″ N, 119°20′16.12″ E). For each cultivar, 30 full ripe fruits (tender and crisp pulps, strong flavor, sweet aftertaste, visually consistent appearance and size, and green or yellow-green peel) were harvested from the four different sides of the crown of each tree, with a total of five trees used for each cultivar. After washing the fruit surface three times using sterile water, the fruits were then quickly frozen in liquid nitrogen, and stored in a −80 °C ultra-low temperature freeezer until further use.

### 2.2. Non-Targeted Metabolomics Analysis

#### 2.2.1. Sample Preparation

Fruit pulp was first cut into pieces of a uniform size of about 1.0 cm × 1.0 cm using a ceramic knife. Then, 200 mg of pulp was placed into a 2 mL Eppendorf tube, before 0.6 mL of 2-chlorophenylalanine (purity ≥ 98.5%, Aladdin, Shanghai, CHN) and methanol (purity ≥ 99.0%, Thermo, Waltham, MA, USA) were added. After mixing for 30 s, 100 mg of glass beads was added and the mixture was placed into a SCIENTZ-48 tissue grinder (SCIENTZ, Ningbo, CHN) at 60 Hz for 90 s. The sample was subsequently treated with ultrasound at room temperature for 15 min before centrifugation at 12,000 rpm at 4 °C for 10 min. Then, 300 μL of supernatant was then filtered using a 0.22 μm membrane and the filtered solution was added to the detection bottle. Finally, 20 μL each sample was mixed with QC (quality control) samples to correct for any deviation in the results of the analysis while the remaining samples were analyzed by LC–MS (liquid chromatography–mass spectroscopy).

#### 2.2.2. Chromatographic Conditions

Chromatographic detection was performed using an UltiMate 3000 liquid chromatographic system (Thermo, Waltham, MA, USA) consisting of a chromatographic column (ACQUITY UPLC^®^ HSS T3; 2.1 mm × 150 mm, 1.8 μm) and an automatic sampler set at a temperature of 8 °C. Gradient elution of analytes was carried out at a flow rate of 0.25 mL/min with 0.1% of formic acid in water (A) and 0.1% of formic acid (LC–MS grade, TCI Shanghai, CHN) in acetonitrile (purity ≥ 99.9%, Thermo, Waltham, MA, USA) (B) or 5 mM of ammonium formate (purity ≥ 99.9%, Sigma, Saint Louis, MO, USA) in water (C) and acetonitrile (D). After equilibrating the column, an injection volume of 2 μL per sample was used, with the following gradient elution procedure: 0~1 min, 2% B/D; 1~9 min, 2~50% B/D; 9~12 min, 50~98% B/D; 12~13.5 min, 98% B/D; 13.5~14 min, 98%~2% B/D; 14~20 min, 2% B-positive model (14~17 min, 2% D-negative model).

#### 2.2.3. Mass Spectrometry Conditions

For mass spectrometry analysis, a Thermo Q Exactive HF-X (Thermo, Waltham, MA, USA) mass spectrometer was used, with positive and negative ion spray voltages of 3.50 kV and 2.50 kV, respectively, as well as a sheath gas and an auxiliary gas flow rates of 30 arb and 10 arb, respectively. The capillary temperature was 325 °C while the full scanning was carried out at a resolution of 60,000, using a scanning range of 81~1000. The secondary cracking was carried out in HCD (high-energy collision dissociation) mode, using a collision voltage of 30 eV, and any unnecessary MS/MS information was removed by dynamic exclusion.

#### 2.2.4. Data Analysis

For data analysis, the different cultivars were divided into four groups labelled as C, T, H, and Z, with each having four biological replicates. The total ion flow diagrams of all samples in positive and negative ion modes were subjected to baseline filtering, peak identification, integration, retention time correction, peak alignment, and normalization by Progenesis QI in order to obtain a data matrix of the retention times, the mass charge ratios (*m*/*z*), and the peak intensities. After importing this data matrix into SIMCA software, PCA (principal component analysis) and PLS-DA (partial least squares discriminant analysis) were performed. Based on the *VIP* (variable importance in projection) scores for the first principal component of the OPLS-DA (orthogonal projections to latent structures discriminant analysis), DAMs in different samples were screened using the criteria of *VIP* value > 1.0 and *p* value < 0.05 [27,28]. Metabolites were mainly identified based on their chromatographic peak retention times and their mass-to-charge (*m*/*z*) ratios, as available in the public databases provided with the Progenesis QI software (http://www.hmdb.ca/ (accessed on 18 January 2021) and http://www.lipidmaps.org/ (accessed on 18 January 2021)) and BioNovoGene (Suzhou, CHN). Metabolic pathways of DAMs were also investigated using the KEGG database (http://www.genome.jp/KEGG/pathway.html (accessed on 18 January 2021 )).

### 2.3. RNA Sequencing

#### 2.3.1. Total RNA Extraction and Detection

Total RNA was extracted from fruit of *C. album* using the E.Z.N.A.™ Plant RNA Kit (Omega, Norcross, GA, USA), and its quality was examined using agarose gel electrophoresis to check for DNA contamination and confirm the integrity of RNA samples. The purity of the samples was then determined by TU-1810 spectrophotometer (Puxi, Beijing, CHN), with the quality eventually assessed using an Agilent 2100 bioanalyzer (Agilent Technologies, Santa Clara, CA, USA).

#### 2.3.2. Library Preparation for RNA-Seq

Illumina’s NEB Next^®^ UltraTM RNA Library Prep Kit (NEB, Ipswich, MA, USA) was used to construct the RNA library. mRNA with polyA tails was first enriched by Oligo (dT) magnetic beads, before fragmentation with divalent cations present in the NEB Fragmentation Buffer. Using fragmented mRNA as template and random oligonucleotides as primers, the first cDNA strand was then synthesized in a M-MuLV reverse transcriptase system. After degrading the RNA strand with RNaseH, the second cDNA strand was synthesized from dNTPs in a DNA polymerase I system. The purified double-stranded cDNA was subsequently repaired at both ends, before adding a tail and the sequencing connectors. cDNA with length of about 250~300 bp were screened with AMPure XP beads for PCR amplification and purified again to obtain the library, which was quantified using a Qubit2.0 Fluorometer. Inserts of appropriate sizes were used to construct libraries, which were diluted to 1.5 ng/μL and subjected to quality control using an Agilent 2100 bioanalyzer. qRT-PCR (quantitative real-time PCR) was then performed to accurately quantify the DNA concentration in the library. Eventually, different libraries were pooled according to the requirements for Illumina-Hiseq sequencing to generate 150 bp paired-end reads.

#### 2.3.3. Data Analysis

After import into CASAVA, the sequenced reads were converted to fastq format and clean data were obtained by removing adapter and low-quality sequences. The Q20 and Q30 scores, along with the GC content of clean data, were calculated. DESeq2 software was used for differential expression analysis between two samples to screen for DEGs. For this purpose, adjusted *p* values and |log_2_^foldchange^| were used to set the threshold for significant differential expression. GO (Gene Ontology) and KEGG (Kyoto Encyclopedia of Genes and Genomes) pathway enrichment analyses were performed by clusterProfiler.

#### 2.3.4. Identification of MBW (MYB- bHLH-WD40) Complex Genes from Transcriptome Data

The MBW complex is composed of MYB, bHLH and WD40, and regulates many biological and physiological processes in plants. To analyze its potential role in the flavonoid synthesis of *C. album*, the *MYB*, *bHLH*, and *WD40* genes were retrieved from our transcriptome data. TBtools were then used to align the sequences of these screened *MYB*, *bHLH*, and *WD40* genes with corresponding gene sequences that have been demonstrated to regulate flavonoid synthesis in other plant species, including *Arabidopsis* [29,30], *P. pyrifolia* [31], *Solanum lycopersicum* [32,33], *Narcissus tazetta* [34], *Chrysanthemum morifolium* [35], *P. bretschneideri* [36], *Populus tremula* × *tremuloides* [37], *Ginkgo biloba* [38,39], *Plagiochasma appendiculatum* [40], *Punica granatum* [41], *Camellia sinensis* [42], etc., to identify candidate gene members. Finally, candidate genes were further screened based on their fold change among samples (≥2.0 or ≤0.5) and abundance (FPKM (fragments per kilobase million) ≥ 10.0).

#### 2.3.5. qRT-PCR Verification

The expression of eighteen selected DEGs, including six *MYB* transcription factors, four *bHLH* transcription factors, and eight functional genes involved in flavonoid biosynthesis (i.e., *FLS*, *CCoAOMT*, *CHI*, *C4H*, *DFR*, *LAR,* and *C3′H*), were verified using qRT-PCR analysis by using *ACTB7* gene of *C. album* as internal reference. TransStart^®^ Top Green qPCR SuperMix (Transgen Biotech, Beijing, CHN) was used for qRT-PCR with the following components: 10 μL of 2 × TransStart^®^ Top Green qPCR SuperMix, 0.4 μL of Passive Reference Dye (50×), 0.2 μM of each primer, 0.5 μM of cDNA template, and ddH_2_O to make up to a final volume of 20 μL. The primer sequences used for amplifying the selected genes are shown in supplemental data Table S1. In addition, the cDNA of different samples was mixed in equal amounts, and then diluted to create a concentration gradient as follows in order to obtain the standard curve: 10^−1^, 40^−1^, 160^−1^, and 640^−1^. An appropriate annealing temperature was also selected according to the amplification efficiency and the qRT-PCR amplification was eventually performed under the following conditions: pre-denaturation at 94 °C for 30 s; and 40 cycles of denaturation at 94 °C for 10 s, annealing for 15 s, and extension at 72 °C for 10 s. The samples were kept at 94 °C for 15 s and then 60 °C for 15 s, before raising the temperature to 94 °C at a rate of 0.11 °C/s for 15 s, to draw the melting curve, with the relative expression of the screened genes in different samples detected by Roche LightCycler480 (Roche, Rotkreuz, Switzerland). Microsoft Excel 2016 and SPSS 19.0 were used for statistical analysis.

## 3. Results

### 3.1. Quality Control (QC) Analysis of Metabolome Data

**Figure 1:**
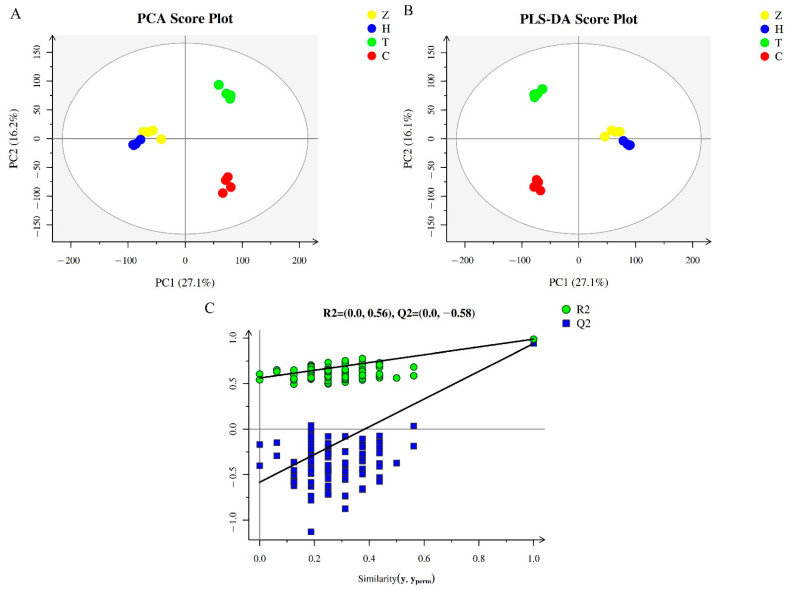
QC analysis results of metabolomics of different *C. album* cultivars. (**A**) PCA score plot for the 21,438 metabolites found in this study. (**B**) PLS-DA score and permutation test plots of the total 21,438 metabolites. (**C**) Displacement inspection result diagram. The yellow, blue, green, and red dots represent *C. album* cv. ‘Zilaiyuan’ (Z), ‘Huiyuan’ (H), ‘Tanxiang’ (T), and ‘Changying’ (C), respectively. The abscissa and ordinate represent the first and the second principal components, respectively.

According to the chromatograms in the LC–MS (supplemental data Figure S1), the sample signals were strong and the peak resolutions were high, indicating that the stability of the LC–MS detection system was good and the data were reliable. PCA reflects the original state of metabolomics data, and can be used to visualize the data characteristics and variations among different cultivars. According to the PCA score plot analysis results (Figure 1A), the *R*^2^*_X_* value was found to be 0.541, indicating that the composition and concentration of metabolites in fruits from the different cultivars varied a lot. Moreover, the four biological replicates of the same cultivar were clustered together, indicating that the metabolomic data were repeatable and very reliable. In particular, Z and H clustered relatively close to each other, with even some partial overlap, indicating that the metabolite composition in the fruits of the ‘Huiyuan’ and ‘Zilaiyuan’ cultivars was quite similar. The metabolomics data for the cultivars T and C were distributed in the first quadrant and fourth quadrant. respectively, thereby suggesting that the metabolic components of ‘Changying’ and ‘Tanxiang’ fruits differed a lot.

To better distinguish the metabolomes of the four *C. album* cultivars, a PLS-DA was applied. The results of the PLS-DA scores and permutation tests are shown in Figure 1B. The results were similar to those of PCA analysis, with Z and H samples clustering close together but far away from the other cultivars, thereby again indicating that the metabolic components of ‘Zilaiyuan’ and ‘Huiyuan’ fruits were similar. The model coefficients *Q*^2^ and *R*^2^*_Y_*values for different cultivars were both higher than 0.93 (0.939 and 0.987, respectively), indicating that the model had good prediction ability and was suitable for the data analysis. Through the permutation test, it was found that the *Q*^2^ point of the model from left to right was lower than the original *Q*^2^ point at the rightmost end, while the intercept of the *Q*^2^ regression line was −0.58 (Figure 1C), which again confirmed that the model was very reliable.

### 3.2. Screening of Differentially Accumulated Metabolites (DAMs) among Fruits from Different C. album Cultivars

By using a *VIP* value of > 1.0 as a criterion, we identified a total of 87 DAMs among the four *C. album* cultivars (*p* < 0.05). These DAMs included metabolites belonging to flavonoids, amino acids, organic acids, sugars, plant hormones, and polyphenols, and so on (Table 1, supplemental data Table S2). After raising the criterion to a *VIP* value of > 1.5, only 41 DAMs remained. Based on OPLS-DA, the pairwise comparison of DAMs between varieties further showed that there were 71, 33, and 72 DAMs between C and H, C and T, and C and Z, respectively, while 52 and 51 different DAMs were identified between T and H, and T and Z, respectively. Moreover, 29 types of DAMs were identified between H and Z. The results indicated that metabolome difference was the largest between ‘Changying’ and ‘Zilaiyuan’, and the smallest between ‘Huiyuan’ and ‘Zilaiyuan’.

Significant differences in the contents of alanine and genistein were found among all the four cultivars. The results of the pairwise comparisons showed that the content of 1-*O*-galloyl-beta-D-glucose, epicatechin, kaempferol, norsanguinarine, and salicylic acid differed significantly only between ‘Changying’ and ‘Huiyuan’, and the L-ribulose content differed greatly only between ‘Changying’ and ‘Zilaiyuan’. Interestingly, a series of some unreported metabolites, including qing hau sau, genistin, (+)-pinoresinol, fraxetin, and eugenol, were also identified in the fruits of *C. album*.

### 3.3. KEGG Pathway Enrichment Analysis of DAMs and Screening of DAFs (Differentially Accumulated Flavonoids)

**Figure 2:**
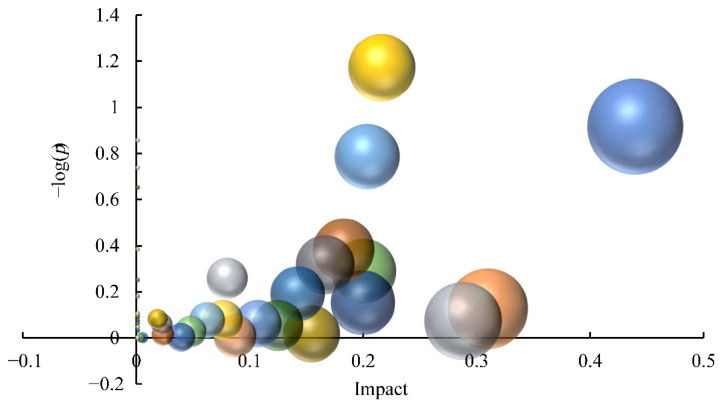
Influence of differential metabolites on the metabolic pathways of distinct *C. album* cultivars. Each dot represents a metabolic pathway. Ordinate values indicate the significance of metabolites enriched in this pathway, with higher values reflecting greater significance levels. The abscissa indicates the influence level of DAMs on the metabolic pathway; the values and dots of highly influenced metabolic pathways are larger.

In total, 39 enriched pathways were identified through KEGG pathway enrichment analysis of DAMs (Figure 2). Pathways with an impact value greater than 0.1 were then screened, which included flavone and flavonol biosynthesis, alpha-linolenic acid metabolism, tryptophan metabolism, flavonoid biosynthesis, lysine biosynthesis, glyoxylate and dicarboxylate metabolism, pentose and glucuronate interconversions, ascorbate and aldarate metabolism, phenylalanine metabolism, terpenoid backbone biosynthesis, the citrate cycle, the pentose phosphate pathway, and arginine and proline metabolism. The impact value of the flavone and flavonol biosynthesis pathway was the highest (0.440), whereas flavonoid biosynthesis enriched the most abundant DAMs, suggesting that DAFs contributed the highest to the metabolome differences among *C. album* cultivars.

In total, 17 DAFs were identified; among them, 12, 7, and 11 of these DAFs were found when comparing C with H, T, and Z, respectively. Similarly, 7 and 7 different DAFs were identified between T, and H and Z, respectively. However, only 6 DAFs were identified between H and Z (Table 1). These results indicated that great differences in flavonoid components existed between ‘Changying’ and ‘Huiyuan’. The identified DAFs between C and H included (2s)-liquiritigenin, apigenin, aromadendrin, epicatechin, kaempferol, leucopelargonidin, luteolin, myricetin, p-coumaroyl quinic acid, quercetin, quercetin 3-o-glucoside, and taxifolin. Of them, the contents of (2s)-liquitigenin and myricetin in H were 16.5- and 13.9-fold of that in C, respectively, suggesting that they were main flavonoids resulting in the metabolome differences between ‘Huiyuan’ and ‘Changying’.

### 3.4. Comparative Transcriptomic Analysis

#### 3.4.1. Overview of the RNA-Seq Data

To further reveal the molecular mechanisms of significant metabolites, especially flavonoids differences between ‘Changying’ and ‘Huiyuan’, a comparative transcriptomic analysis of ‘Changying’ (T_C) and ‘Huiyuan’ (T_H) fruits was performed. As shown in Table 2, the Q20 and Q30 values of all the cDNA libraries were greater than 98% and 93%, respectively, and the base error rate was less than 0.03%. Furthermore, the N50 and N90 values of the transcripts were 2331 bp and 760 bp, respectively, and the N50 and N90 values of genes were 2036 bp and 495 bp, respectively. These transcriptome data for *C. album* were therefore of good quality and satisfied the requirements for subsequent analysis.

#### 3.4.2. Identification and Enrichment Analysis of DEGs

**Figure 3:**
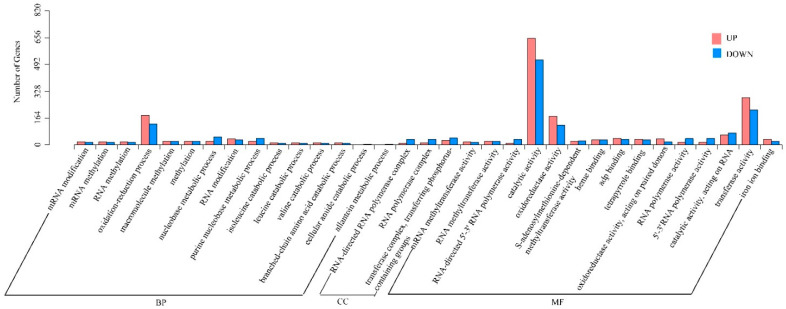
GO classification of upregulated and downregulated DEGs identified between ‘Changying’ and ‘Huiyuan’ fruits. BP: biological process; CC: cellular component; MF: molecular function.

By using a *q* value of < 0.05 and a |log_2_^fold change^| of > 1.0 as thresholds, the DEGseq method was applied to screen for DEGs between ‘Changying’ and ‘Huiyuan’ fruits. Compared with T_H, 3665 DEGs (1762 downregulated and 1903 upregulated) were identified in T_C (supplemental data Table S3). After classifying these DEGs into cellular component (CC), molecular function (MF), or biological process (BP) terms based on GO enrichment analysis (Figure 3), a total of 3060 enriched GO terms were identified. From the aspect of BP, 15 types of biological processes were significantly enriched, with the largest number of DEGs involving in oxidation–reduction processes. From the aspect of MF, 15 GO terms were significantly enriched, including 1172 catalytic activity-related DEGs, as well as more than 300 DEGs involved in transferase activity, oxidoreductase activity, and adenyl nucleoside binding. From the aspect of CC, only three GO terms were identified to be significantly enriched.

KEGG enrichment analysis of DEGs identified a total of 106 enriched pathways, and 15 of them were found to be significantly enriched (*p* < 0.05) (Table 3). The significantly upregulated and downregulated DEGs, along with their corresponding pathways, can be presented as follows:

- 22 upregulated and 22 downregulated DEGs involved in plant hormone signal transduction;
- 9 upregulated and 7 downregulated DEGs involved in carotenoid biosynthesis;
- 14 upregulated and 15 downregulated DEGs involved in phenylpropane biosynthesis;
- 30 upregulated and 3 downregulated DEGs involved in plant pathogen interaction;
- 8 upregulated and 2 downregulated DEGs involved in flavonoid biosynthesis;
- 5 downregulated and 7 upregulated DEGs involved in ABC transport;
- 9 fatty acid elongation related and 14 fatty acid biosynthesis related DEGs, for which 8 and 2 DEGs, respectively, were inhibited;
- 8 genes were found to be inhibited and 19 genes were induced in glycolysis/gluconeogenesis;
- Among the 15 galactose metabolism related DEGs, 5 DEGs were downregulated;
- Stilbenoid, diarylheptanoid, and gingerol biosynthesis contained 1 downregulated gene and 4 upregulated genes;
- Among the 12 and 9 DEGs involved in phenylalanine metabolism and tryptophan metabolism, 7 and 1 DEGs were upregulated, respectively;
- 1 upregulated and 3 downregulated DEGs involved in limonene and pinene degradation;
- 5 out of 6 DEGs involved in sesquiterpenoid and triterpenoid biosynthesis were induced.

It is worth noting that the pathway of flavonoid biosynthesis, which contained significantly enriched DEGs (*p* = 1.94 × 10^−3^), was also greatly enriched by DAMs.

### 3.5. Conjoint Analysis of Metabolomics and Transcriptomics Data

**Figure 4:**
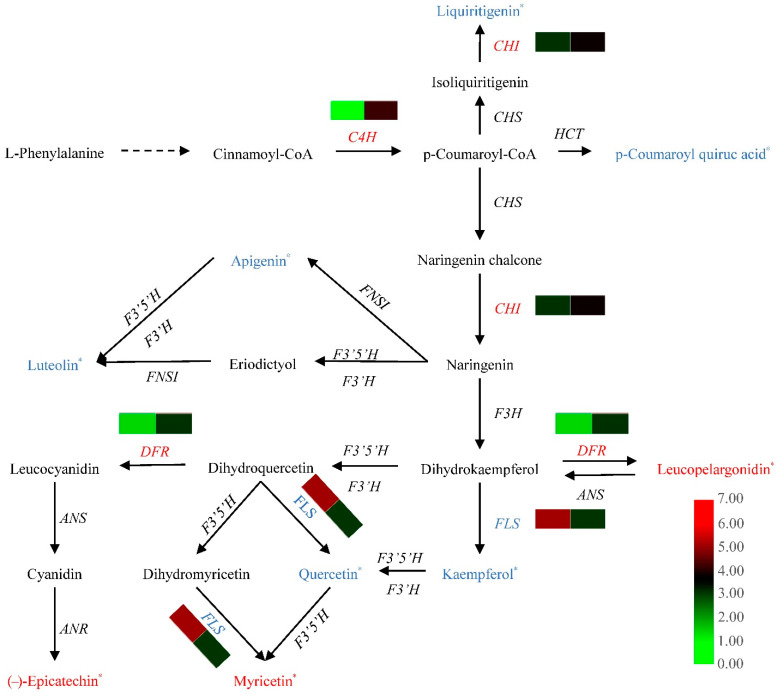
Correlation analysis of flavonoid biosynthesis-related DEGs and DAMs. Different colors in the heatmap indicate different gene expression levels; * indicates that there is a significant difference in the content of the metabolite between the two cultivars. Red indicates that the gene expression level or metabolite content in ‘Huiyuan’ is greater than that in ‘Changying’, and blue indicates that the gene expression level or metabolite content in ‘Huiyuan’ is less than that in ‘Changying’. C4H: cinnamic acid 4-hydroxylase; CHS: chalcone synthase; CHI: chalcone isomerase; HCT: hydroxycinnamoyl-CoA: shikimate hydroxycinnamoyl transferase; FNSI: type I flavone synthase; F3′5′H: flavonoid-3′-5′-hydroxylase; F3′H: flavonoid-3′-hydroxylase; F3H: flavonone-3-hydroxylase; FLS: flavonol synthase; DFR: dihydroflavonol reductase; ANS: anthocyanidin synthase; ANR: anthocyanidin reductase.

Based on the information in the flavonoid biosynthesis pathway (ko00941), Pearson’s correlation analysis of DAFs and DEGs identified between ‘Changying’ and ‘Huiyuan’ fruits was performed (Figure 4). Compared with ‘Changying’, the liquiritigenin, p-coumaroyl quiuc acid, apigenin, luteolin, kaempferol, and quercetin content in ‘Huiyuan’ was significantly lower, while its leucopelargonidin, myricetin, and (−)-epicatechin content was significantly higher. It was found that two *CHIs* showed negative correlation to liquiritigenin (*r* = −0.466, *p* = 0.352). The transcription levels of two *DFRs* were positively correlated with the accumulation of leucopelargonidin (*r* = 0.114, *p* = 0.830). Moreover, the expression levels of two *FLSs* were found to be positively correlated with the kaempferol (*r* = 0.969, *p* = 0.001), quercetin (*r* = 0.991, *p* = 0) and myricetin (*r* = −0.774, *p* = 0.071) content.

### 3.6. Identification and Quantitative Real-Time PCR Verification of Flavonoid Biosynthesis Related Structural Genes and Transcription Factor Genes

**Figure 5:**
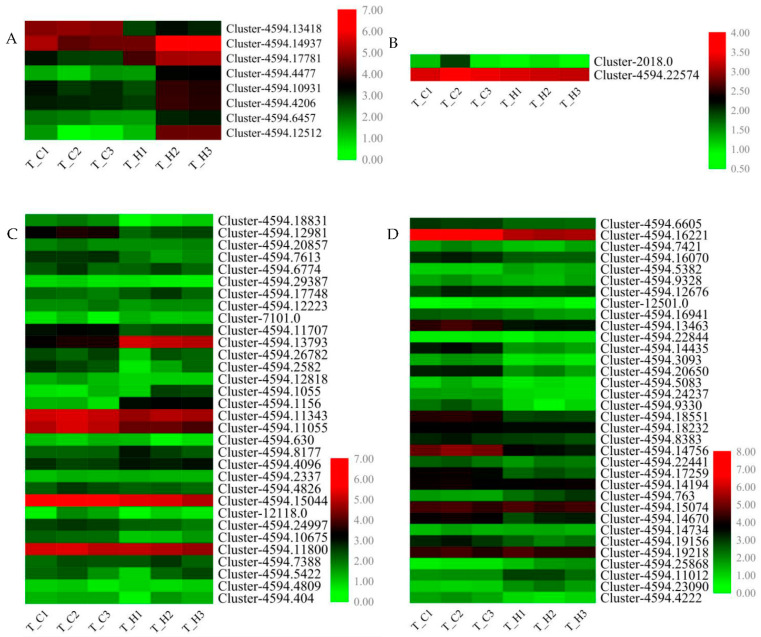
Heat map analysis of FPKM values of related genes involved in flavonoid biosynthesis. (**A**) The map shows the change in FPKM values of selected DEGs in flavonoid biosynthesis in different *C. album* cultivars. (**B**) Heat map showing the changes in FPKM values of selected *WD40* transcription factors in different cultivars of *C. album*. (**C**) Heat map showing the changes in FPKM values of selected *MYB* transcription factors in different cultivars of *C. album*. (**D**) Heat map showing the changes in FPKM values of selected *bHLH* transcription factors in different cultivars of *C. album*. Each column represents a given sample and each row indicates relative expression or FPKM value of the gene written on the right-hand side.

Given the significant contribution of flavonoid metabolism pathway to the fruit metabolome differences in diverse *C. album* cultivars, we further validated the expression of eight flavonoid biosynthesis structural DEGs, *FLS*, *CCoAOMT*, *C3′H*, *DFR*, *CHI*, *C4H,* and two *LARs*, in ‘Changying’ and ‘Huiyuan’ fruits using qRT-PCR (Figure 5A). According to our transcriptome data, the expression level of *FLS* in ‘Changying’ was 4 times higher than that in ‘Huiyuan’, while the expression of *C4H* in ‘Huiyuan’ was 15 times higher than that in ‘Changying’. In addition to structural genes, the flavonoid synthesis is also regulated by regulatory factors, such as the MBW complex, which is comprised of MYB, bHLH, and WD40 proteins [43]. In this study, we identified 59 *MYB* and 57 *bHLH* genes from our transcriptome data (supplemental data Tables S4 and S5). Through homologous alignment analysis, 32 *MYBs* and 34 *bHLHs* were identified as candidate flavonoid biosynthesis-related transcription factors (Figure 5C,D). Among them, 10 *MYBs* and 15 *bHLHs* showed more than 2-fold change between ‘Changying’ and ‘Huiyuan’ fruits. After combining the results of fold change and expression abundance, six and four candidate *MYB* and *bHLH* transcription factors that might regulate flavonoid synthesis in the fruits of *C. album* were finally selected for further qRT-PCR verification (supplemental data Table S6). Besides, 122 *WD40* transcription factors were identified from our transcriptome data (supplemental data Table S7), and through homologous sequence blasting, only two of them were identified as candidate flavonoid biosynthesis related. However, the expression levels of the two *WD40* members did not show significant changes between the two cultivars (Figure 5B).

**Figure 6:**
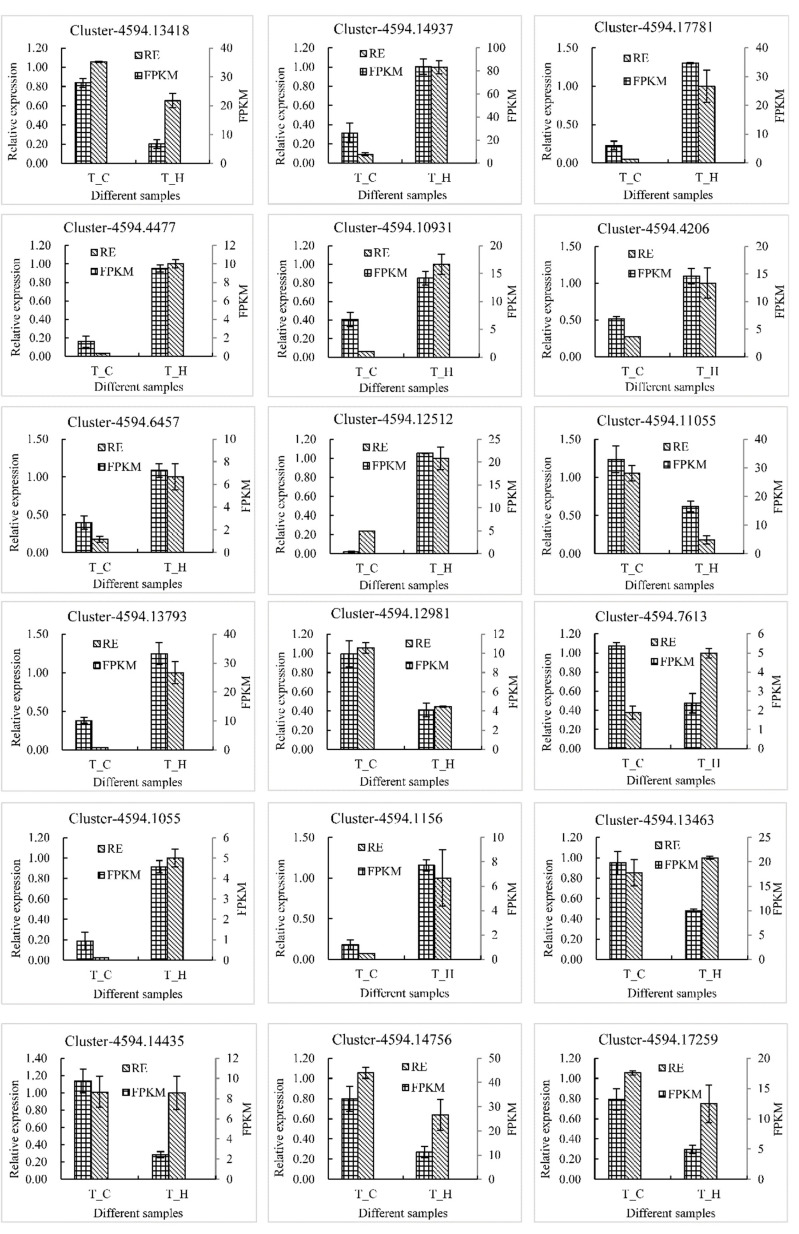
qRT-PCR verification of candidate genes related to flavonoid synthesis. RE and FPKM respectively represent the relative expression and fragments per kilobase million of each gene in different samples. Cluster-4594.13418, Cluster-4594.14937, Cluster-4594.17781, Cluster-4594.4477, Cluster-4594.10931, Cluster-4594.4206, Cluster-4594.6457, and Cluster-4594.12512 represent *FLS*, *CCoAOMT*, *C3′H*, *DFR*, *LAR, CHI*, *LAR*, and *C4H* genes, respectively. Cluster-4594.11055, Cluster-4594.13793, Cluster-4594.12981, Cluster-4594.7613, Cluster-4594.1055, and Cluster-4594.1156 belong to the *MYB transcription factor* gene family, and Cluster-4594.13463, Cluster-4594.14435, Cluster-4594.14756, and Cluster-4594.17259 are different members of the *bHLH transcription factor* gene family.

qRT-PCR analysis of the eight selected flavonoid biosynthetic structural genes showed that the expression of *FLS* gene in the ‘Changying’ cultivar was higher than that in ‘Huiyuan’ cultivar, whereas the expression levels of the other genes, including *CCoAOMT*, *C3’H*, *DFR*, *LAR*, *CHI*, and *C4H* genes in the ‘Huiyuan’ cultivar were more than 3.0 times multiple than ‘Changying’ cultivar. Notably, the expression of *DFR* gene in ‘Huiyuan’ was found to be 32-fold higher than that in ‘Changying’. Among the six *MYB*s, three genes showed higher expression levels in ‘Changying’ and three showed lower expression levels. Notably, the expression levels of two *MYBs* (Cluster-4594.13793 and Cluster-4594.1156) in ‘Huiyuan’ were approximately 31 and 40 times higher than that in ‘Changying’, respectively. The expression of four candidate *bHLHs* showed no significant difference between the two cultivars. Generally, the expression patterns of these genes in different samples were almost consistent with the sequencing results (except Cluster-4594.7613 and Cluster-4594.13463) (Figure 6), confirming that our transcriptome data are reliable.

## 4. Discussion

### 4.1. Metabolomics Analysis Distinguished the C. album Cultivars Well and Can Provide a Basis for the Rational Utilization of C. album

Untargeted metabolomics reflects the comprehensive dynamics of endogenous metabolites in organisms. By comparing the differential metabolites in different *C. album* fruit samples, it is possible to effectively identify varieties or regions, and provide a reference for assessing biological evolution and the identification of genetic relationships [44,45]. In the present study, based on the results of PCA and PLS-DA analyses, the metabolomes of ‘Huiyuan’ and ‘Zilaiyuan’ cultivars were found to be the most similar, which was consistent with their genetic relationship: the ‘Zilaiyuan’ cultivar was a mutant of ‘Huiyuan’ cultivar.

Metabolomics can systematically reveal the metabolic state of organisms affected by genetic or external factors [46]. It has been successfully applied in many crops for the exploration of differential metabolites in a variety of crop germplasms, as well as in studying the mechanism for the formation of certain active ingredients. For example, through the combined analysis of fruit quality indices and metabolomes, Xi et al. found that cyanidin, anthocyanin-3-o-rutin, and paeoniflorin were the main factors responsible for the red color formation in *Prunus armeniaca* [47]. Wang et al. revealed that the increase in abscisic acid, jasmonic acid, and ethylene, and with decrease in auxin and brassinosterol were the main reasons causing *Litchi chinensis* fruit cracking [48]. The purple fruits of *Passiflora edulis* were rich in flavonols, anthocyanins, and flavanols, while flavonoid and flavonoid glycosides accumulated a lot in the yellow fruits [49]. Ghisoni et al. reported the differences in phenols and sterols of different virgin olive cultivars, and explored the possibility of using the most abundant compounds to distinguish different cultivars and the same cultivar cultivated in different regions [50]. Moreover, the correlation between flavonoid content and virgin olive cultivars was also reported [51]. Therefore, it can be concluded that metabolomics has been and will continue to be of great significance for revealing traits that reflect crop quality as well as the mechanisms underlying the production of active ingredients.

### 4.2. Flavonoid Accumulation Is a Key Determinant of the Metabolome Differences among C. album Cultivars

Flavonoids and polyphenols have been shown to be the most important functional components in *C. album* fruits, but their contents are highly variable among different cultivars [11]. By comparing the different metabolites in the fruits of ‘Changying’, ‘Huiyuan’, ‘Tanxiang’, and ‘Zilaiyuan’, a large number of DAMs involved in flavonoids or polyphenols metabolism were found, indicating that flavonoids or polyphenols contributed largely to the metabolome differences between these cultivars. Moreover, KEGG pathway enrichment analysis of DEGs identified between ‘Changying’ and ‘Huiyuan’ also revealed the significant enrichment of flavonoid biosynthesis pathway, confirming that the flavonoid accumulations in different *C. album* fruits varied considerably. Among the flavonoids, apigenin has been reported to have the ability to protect the liver and inhibit tumor cells [52], leucopelargonidin can alleviate diabetes [53], quercetin can resist oxidation and inflammation [54], (−)-epigallocatechin can reduce uric acid activity [55], aromadendrin can inhibit T cell activity [56], myricetin can resist oxidation and protect nerves and (2s)-liquiritigenin can inhibit tumors [57,58]. Since these active components were specifically enriched, they were inferred to be closely linked to the pharmacological properties of *C. album* fruits. In addition, in this study, the presence of qing hau sau, genistin, (+)-pinoresinol, fraxetin, and eugenol in the fruits of *C. album* was reported for the first time; this should be confirmed or become a focus of the future study of the medicinal value of *C. album*.

### 4.3. Transcription of Flavonoid Biosynthetic Structural Genes and Transcription Factor Genes Contributed Greatly to the Metabolome Differences among Fruits of Different C. album Cultivars

The flavonoid biosynthesis pathway plays an important role in plant flavonoid accumulation. In our study, many flavonoid biosynthetic structural genes were identified as DEGs by transcriptome data, which was further confirmed by qRT-PCR. Among them, *FLS* was highly expressed in the ‘Changying’ cultivar, whereas the expression levels of *CCoAOMT*, *C3’H*, *DFR*, *LAR*, *CHI*, and *C4H* genes in the ‘Huiyuan’ cultivar were higher than that in the ‘Changying’ cultivar. All these selected genes have been shown to play important regulatory roles in flavonoid synthesis in many plants [59]. The differential expression of these genes in different cultivars of *C. album* might satisfactorily explain the variations in flavonoid accumulation.

As important components of the MBW complex, MYB and bHLH represent key transcription factors regulating plant flavonoid synthesis. Reports have shown that *Pyrus bretschneideri PbMYB10b* was an activator of the anthocyanin and procyanidin pathway and that *PbMYB9* functioned in activating of the flavonol biosynthesis [36]. The overexpression of *McMYB12a* and *McMYB12b* in *Malus* crabapple increased the expression of flavonoid biosynthesis genes and promoted the accumulation of procyanidins and anthocyanins [60]. *MdMYB3* positively regulated the expression of *CHS*, *CHI*, *UFGT,* and *FLS* in *M.* × *domestica* [61]. *FhMYB5* upregulated the expression of *DFR* and *LDOX* in *F. hybrida* [62]. *CmMYB8* negatively regulated flavonoid synthesis in chrysanthemum [35]. These results showed that *MYB* played important roles in regulating plant flavonoid biosynthesis. In our study, by homologous sequence alignment using reported flavonoid biosynthesis-related *MYB* sequences, 32 candidate *MYBs* regulating flavonoid biosynthesis of *C. album* were identified. Eventually, six candidate *MYB* transcription factors were obtained by screening the differential multiples and expression abundances, including cluster-4594.12981 and cluster-4594.11055, which were differentially expressed with a fold-change of 2.3 and 5.8 between the two cultivars, had the highest homology with *MYB4* and *MYB60* of *A. thaliana*, respectively. *AtMYB4* has been proven to play a dual role in flavonoid synthesis, and *AtMYB60* is a transcription inhibitor of flavonoid synthesis [29,63]. These two members were significantly differentially expressed in different cultivars of *C. album* which present differential flavonoid components and contents, indicating that these *MYB* transcription factors might regulate flavonoid synthesis in *C. album*.

bHLH has also been proved to be an important transcription factor affecting plant flavonoid synthesis. *LcbHLH92* negatively regulated the transcriptional levels of *ANS* and *ANR* in *Leymus chinensis*, and *CcbHLH6*-*1* significantly upregulated the activities of *F3H* and *DFR* promoters of *Centaurea cyanus* [64,65]. Similarly, both *P. appendiculatum* and *Vitis vinifera bHLH1* gene functioned in activating flavonoid synthesis [40,66]. In our study, we identified 34 candidate flavonoid biosynthesis-related *bHLH* genes. Among them, 15 *bHLHs* showed more than two-fold changes between ‘Changying’ and ‘Huiyuan’ fruits, indicating that they might play important roles in the flavonoids biosynthesis in *C. album* and that their functions need to be studied further.

## 5. Conclusions

In this study, an untargeted metabolomics technique was successfully applied to distinguish the metabolome difference in the fruits of four different *C. album* cultivars. The results showed that the differential accumulations of flavonoids contributed majorly to the various metabolome compositions of fruits from different *C. album* cultivars. In agreement with our metabolome results, the flavonoid biosynthesis pathway was significantly enriched by DEGs encoding flavonoid biosynthesis-related structural proteins and transcription factors. Our study will be very helpful in clarifying the underlying mechanism of the differences in metabolite composition and accumulation among different *C. album* cultivars.

## Figures and Tables

**Table 1:** Differential metabolites in the comparison of different cultivars.

Metabolites	H vs. C		T vs. C		Z vs. C		H vs. T		Z vs. T		Z vs. H	
	VIP	*p*	VIP	*p*	VIP	*p*	VIP	*p*	VIP	*p*	VIP	*p*
(−)-Epigallocatechin *	1.064	0.061	0.175	0.885	0.825	0.312	1.325	0.030	1.266	0.030	0.950	0.312
(+)-Pinoresinol	1.064	0.061	0.922	0.112	1.267	0.030	1.371	0.030	1.491	0.030	0.763	0.194
(1S,2R,4S)-(-)-Bornyl acetate	0.664	0.194	0.544	0.470	1.469	0.030	0.431	0.665	1.494	0.030	1.593	0.030
(2S)-Liquiritigenin *	1.125	0.021	0.479	0.312	1.198	0.021	1.146	0.021	1.252	0.021	NA	NA
(S)-Abscisic acid	0.864	0.030	1.105	0.112	1.351	0.030	0.364	0.665	1.403	0.030	1.576	0.030
10-Hydroxydecanoic acid	1.188	0.030	1.627	0.030	1.345	0.030	1.397	0.030	1.271	0.030	0.070	0.665
1-Dehydro-[6]-gingerdione	1.295	0.030	1.251	0.030	1.220	0.030	1.083	0.112	0.915	0.194	0.859	0.112
1H-Indole-3-acetamide	1.357	0.030	1.399	0.030	1.346	0.030	1.364	0.030	1.417	0.030	0.972	0.312
1-*O*-Galloyl-beta-D-glucose	1.051	0.030	0.154	0.885	0.963	0.061	0.753	0.030	0.734	0.030	0.950	0.112
2,3,4,4,6-Peptahydroxychalcone 4-*O*-glucoside	1.278	0.021	1.121	0.194	1.377	0.021	1.269	0.021	1.365	0.021	NA	NA
4-Acetamidobutanoic acid	1.262	0.030	1.295	0.030	1.267	0.030	0.571	0.312	0.049	0.470	1.016	0.112
4-Methoxyflavanone	0.601	0.312	0.578	0.885	1.449	0.030	0.391	0.665	1.339	0.030	0.347	0.885
6-Phosphogluconic acid	1.334	0.021	NA	NA	0.938	0.021	1.336	0.021	1.067	0.021	NA	NA
9(S)-HPOT	1.260	0.030	0.255	1.000	1.358	0.030	1.046	0.030	1.126	0.030	1.415	0.030
Acacetin	1.259	0.030	0.709	0.665	1.399	0.030	0.317	1.000	1.206	0.030	0.805	0.312
Alanine	1.353	0.030	1.650	0.030	1.210	0.030	1.393	0.030	1.449	0.030	1.574	0.030
Alpha-Linolenic acid	1.176	0.030	1.340	0.061	1.398	0.030	0.404	0.312	1.194	0.030	1.104	0.030
Apigenin *	1.296	0.030	1.477	0.030	1.414	0.030	0.981	0.112	1.259	0.030	0.950	0.194
Apocynin	1.271	0.030	1.141	0.061	1.343	0.030	1.194	0.030	1.187	0.061	0.284	0.665
Aromadendrin *	1.347	0.030	1.637	0.030	1.427	0.030	1.198	0.030	0.610	0.470	0.989	0.112
Ascorbate	1.229	0.030	0.600	0.470	1.121	0.030	1.106	0.030	1.121	0.030	1.484	0.030
Astragalin *	0.279	0.301	0.633	0.453	1.452	0.030	0.519	0.301	1.358	0.021	1.308	0.021
Azelaic acid	1.319	0.030	0.844	0.312	1.418	0.030	0.892	0.194	0.935	0.312	0.989	0.112
Betonicine	1.364	0.030	1.301	0.061	1.352	0.030	1.387	0.030	1.216	0.061	0.729	0.665
Catechol	1.268	0.030	0.804	0.312	1.399	0.030	0.029	0.312	0.230	0.312	1.273	0.061
Chlorogenic acid *	0.105	0.885	1.429	0.030	1.333	0.030	1.294	0.030	0.363	0.194	1.190	0.030
Chrysoeriol	1.339	0.030	1.513	0.030	1.316	0.030	0.260	1.000	0.942	0.194	0.099	0.665
Cinnamaldehyde	1.346	0.021	1.434	0.030	1.451	0.021	1.125	0.021	1.210	0.021	NA	NA
cis-Aconitic acid	0.602	0.312	1.254	0.061	1.235	0.030	1.029	0.061	0.973	0.061	1.397	0.030
Citramalic acid	1.182	0.030	0.130	1.000	0.804	0.194	1.148	0.030	0.803	0.112	1.593	0.030
Citric acid	1.341	0.030	0.043	0.665	1.468	0.030	1.261	0.030	1.437	0.030	0.247	1.000
Costunolide	1.164	0.030	0.871	0.194	1.099	0.030	1.134	0.030	1.153	0.030	1.396	0.030
Cucurbitacin E	1.247	0.021	NA	NA	1.240	0.021	1.270	0.021	1.261	0.021	1.450	0.030
D-Maltose	1.125	0.021	NA	NA	1.283	0.021	1.145	0.021	1.304	0.021	1.570	0.030
Ellagic acid	1.211	0.030	1.426	0.030	1.279	0.030	0.410	0.312	0.008	1.000	1.388	0.030
Epicatechin *	1.209	0.030	0.471	0.885	0.981	0.112	1.145	0.061	0.078	0.312	0.104	0.312
Estragole	1.151	0.030	1.044	0.112	1.278	0.030	0.524	0.665	0.757	0.470	1.313	0.030
Eugenol	1.374	0.021	0.222	0.312	1.480	0.021	1.081	0.069	1.163	0.069	NA	NA
Fraxetin	0.978	0.030	1.377	0.030	1.203	0.030	1.220	0.030	0.371	0.470	0.023	1.000
Fructose-1P	1.272	0.021	0.300	0.665	1.353	0.021	1.222	0.021	1.335	0.021	NA	NA
gamma-Aminobutyric acid	1.281	0.030	1.414	0.030	1.191	0.030	1.378	0.030	1.407	0.030	0.763	0.194
Garbanzol *	0.507	0.312	1.523	0.030	0.884	0.312	1.100	0.061	1.218	0.061	1.217	0.030
Genistein	1.173	0.030	1.440	0.030	1.260	0.030	1.355	0.030	1.481	0.030	1.571	0.030
Genistin	0.799	0.194	1.448	0.030	1.349	0.030	1.156	0.030	0.353	0.312	1.104	0.030
Glycylleucine	1.236	0.030	1.358	0.030	1.393	0.030	0.690	0.312	1.265	0.030	1.576	0.030
Herniarin	1.280	0.030	1.042	0.194	1.414	0.030	0.312	1.000	0.643	0.312	1.397	0.030
Hydroxypyruvic acid	1.302	0.021	0.653	0.665	1.403	0.021	1.252	0.021	1.346	0.021	NA	NA
Indoleacetic acid	1.369	0.030	1.649	0.030	1.383	0.030	1.385	0.030	1.346	0.030	0.396	0.47
Isopentenyl pyrophosphate	1.360	0.021	1.421	0.030	1.448	0.021	1.168	0.021	1.275	0.021	NA	NA
Kaempferide *	0.988	0.021	0.830	0.312	0.610	0.453	0.504	0.301	0.415	0.301	1.088	0.021
Kaempferol *	1.342	0.030	0.118	0.312	0.125	0.470	0.796	0.194	0.252	0.885	0.291	1.000
Lamiide	1.353	0.030	0.255	0.665	1.482	0.030	0.467	0.312	0.743	0.312	0.660	0.665
L-Arginine	0.875	0.030	1.607	0.030	0.784	0.665	1.127	0.030	1.101	0.030	NA	NA
L-Asparagine	1.207	0.021	1.394	0.030	1.301	0.021	1.367	0.021	1.470	0.021	NA	NA
L-Cystine	1.323	0.021	0.665	0.470	1.425	0.021	0.941	0.021	1.012	0.021	NA	NA
Leucopelargonidin *	1.123	0.030	NA	NA	1.288	0.030	1.223	0.030	1.357	0.030	0.245	0.885
L-Histidine	1.144	0.021	1.207	0.112	1.233	0.021	1.151	0.021	1.238	0.021	NA	NA
Limonene-1,2-diol	1.174	0.021	0.647	0.470	1.265	0.021	1.200	0.021	1.291	0.021	0.859	0.112
L-Isoleucine	1.221	0.021	0.903	0.194	1.473	0.021	1.224	0.021	1.514	0.021	0.888	0.194
L-Leucine	1.231	0.021	1.444	0.030	1.326	0.021	1.220	0.021	1.312	0.021	0.173	0.665
L-Lysine	0.858	0.112	1.453	0.030	1.163	0.030	1.148	0.030	0.339	0.665	1.008	0.061
L-Ribulose	0.900	0.194	0.903	0.194	1.032	0.030	0.442	0.665	0.752	0.112	NA	NA
Luteolin *	1.141	0.030	0.182	0.665	0.783	0.194	1.291	0.030	0.580	0.885	0.389	1.000
Malvidin 3-glucoside	1.412	0.030	1.662	0.030	1.510	0.030	0.617	0.312	1.355	0.030	1.498	0.030
meso-2,6-Diaminoheptanedioate	1.368	0.030	1.585	0.030	1.384	0.030	1.396	0.030	1.453	0.030	0.336	0.470
myo-Inositol	1.236	0.030	0.591	0.312	1.388	0.030	1.321	0.030	1.465	0.030	0.247	1.000
Myricetin *	1.102	0.021	0.789	0.312	1.172	0.021	1.129	0.021	1.233	0.021	NA	NA
Naringenin 7-*O*-beta-D-glucoside	1.299	0.030	1.343	0.061	1.075	0.030	1.339	0.030	1.320	0.030	1.491	0.030
Norsanguinarine	1.159	0.030	0.046	0.665	1.034	0.061	1.015	0.061	0.724	0.194	1.138	0.312
Palmitoleic acid	1.354	0.030	0.610	1.000	1.460	0.030	1.114	0.030	1.200	0.030	0.973	0.030
p-Coumaroyl quinic acid *	1.310	0.030	1.279	0.030	1.350	0.030	1.147	0.061	1.537	0.030	1.079	0.030
Pelargonic acid	1.219	0.030	1.081	0.112	1.164	0.030	1.299	0.030	1.322	0.030	1.284	0.030
Perillic acid	1.002	0.112	1.306	0.061	0.988	0.312	1.204	0.030	1.184	0.061	0.010	0.665
Procyanidin B2	1.123	0.030	1.312	0.030	0.968	0.030	0.577	0.312	1.056	0.112	1.491	0.030
Pulegone	1.311	0.030	0.485	0.665	1.255	0.030	1.291	0.030	1.230	0.030	0.441	0.885
Qing Hau Sau	1.078	0.030	1.089	0.061	1.182	0.030	1.173	0.030	1.268	0.030	0.396	0.470
Quercetin *	1.326	0.030	1.660	0.030	1.239	0.030	0.675	0.112	0.562	0.885	0.432	0.665
Quercetin 3-*O*-glucoside *	1.019	0.014	1.634	0.030	1.396	0.030	1.145	0.030	0.406	0.665	0.347	0.885
Raucaffricine	1.366	0.030	1.544	0.030	1.431	0.030	1.307	0.030	1.142	0.061	1.348	0.030
Salicylic acid	1.054	0.030	0.212	1.000	0.987	0.112	0.981	0.030	0.919	0.112	0.972	0.312
Silibinin	1.345	0.030	0.792	0.312	0.988	0.112	1.343	0.030	0.900	0.312	0.403	0.47
Syringin	1.181	0.021	1.482	0.030	1.272	0.021	1.349	0.021	1.451	0.021	NA	NA
Taxifolin *	1.136	0.030	0.592	0.312	1.287	0.030	0.411	1.000	0.736	0.194	1.474	0.030
trans-Cinnamate	1.295	0.030	1.438	0.030	1.403	0.030	0.509	0.665	0.748	0.112	1.316	0.030
Uridine	1.339	0.021	1.489	0.030	1.443	0.021	1.176	0.021	1.265	0.021	NA	NA
Vaccenic acid	1.095	0.021	0.988	0.061	1.179	0.021	1.328	0.021	1.429	0.021	NA	NA
Xanthyletin	1.184	0.030	0.572	0.665	1.345	0.030	0.993	0.061	1.144	0.030	1.085	0.112

Note: the underlined values indicate that the corresponding metabolite was significantly different between the two cultivars, when using criteria *VIP* > 1.0 and *p* < 0.05. * indicates flavonoids. NA indicates the values were not detected.

**Table 2:** Quality analysis of RNA sequencing data for the ‘Changying’ (T_C) and ‘Huiyuan’ (T_H) cultivars.

Sample	Raw Reads	Clean Reads	Clean Bases	Error (%)	Q20 (%)	Q30 (%)	GC Content (%)
T_C1	22,741,237	21,927,175	6.58 G	0.02	98.16	94.26	44.30
T_C2	22,753,116	21,910,412	6.57 G	0.03	98.03	93.99	44.17
T_C3	22,262,027	21,498,628	6.45 G	0.02	98.22	94.48	44.39
T_H1	22,897,426	22,263,553	6.68 G	0.03	98.01	93.96	44.64
T_H2	23,830,992	22,859,162	6.86 G	0.02	98.08	94.17	44.61
T_H3	22,397,559	21,655,410	6.50 G	0.02	98.14	94.29	44.79

**Table 3:** KEGG enrichment analysis of DEGs identified between ‘Changying’ and ‘Huiyuan’ fruits.

KEGG Pathways	ID	Input Number	Background Number	*p*-Value
Plant hormone signal transduction	ko04075	44	213	2.29 × 10^−5^
Carotenoid biosynthesis	ko00906	16	41	2.87 × 10^−5^
Phenylpropanoid biosynthesis	ko00940	29	132	2.30 × 10^−4^
Plant–pathogen interaction	ko04626	33	177	1.14 × 10^−3^
Flavonoid biosynthesis	ko00941	10	29	1.94 × 10^−3^
Fatty acid elongation	ko00062	9	25	2.55 × 10^−3^
Fatty acid biosynthesis	ko00061	14	54	2.64 × 10^−3^
ABC transporters	ko02010	12	45	4.30 × 10^−3^
Glycolysis/Gluconeogenesis	ko00010	27	158	8.49 × 10^−3^
Galactose metabolism	ko00052	15	70	8.62 × 10^−3^
Stilbenoid, diarylheptanoid and gingerol biosynthesis	ko00945	5	11	0.011
Phenylalanine metabolism	ko00360	12	53	0.013
Tryptophan metabolism	ko00380	9	37	0.020
Limonene and pinene degradation	ko00903	4	10	0.032
Sesquiterpenoid and triterpenoid biosynthesis	ko00909	6	22	0.036

## Data Availability

The data supporting the results of this study are included in the present article.

## Supplementary Materials

The following supporting information can be downloaded at: https://www.mdpi.com/article/10.3390/foods11162527/s1, Table S1: List of qRT-PCR primers used in the study; Table S2: Differential metabolites when comparing different cultivars; Table S3: DEG annotation of T_C compared with T_H; Table S4: Identification of *MYB* genes from transcriptomic data of *Canarium album*; Table S5: Identification of *bHLH* genes from transcriptomic data of *Canarium album*; Table S6: The gene ID numbers and FPKM values of selected *MYB* and *bHLH* transcription factors; Table S7: Identification of *WD40* genes from transcriptomic data of *Canarium album*; Figure S1: Chromatograms for the LC-MS results of fruits of four *Canarium album* cultivars.
