# Supplementary material for: Integrated Metabolomic and Transcriptomic Analysis Reveals Differential Flavonoid Accumulation and Its Underlying Mechanism in Fruits of Distinct Canarium album Cultivars
Source: Foods. 2022 Aug 21;11(16):2527. doi: 10.3390/foods11162527 (PMC9407539; doi:10.3390/foods11162527)
Supplement: Supplementary file 1 [file foods-11-02527-s001.zip › Table S1. List of qRT-PCR primers used in the study.pdf]

**Table S1**. List of qRT-PCR primers used in the study

| Gene_ID | Primer sequence (5′-3′) | | Product size/bp | Description |
|---|---|---|---|---|
| | Upstream | Downstream | | |
| Cluster-4594.13418 | TGGGCAGGAATCTTGTTG | TGTCAATCGGTCAGGCTAC | 90 | Flavonol synthase |
| Cluster-4594.14937 | GTAGCCTGTGTAGACGCCAA | TATCCAAGAGAGCCTGAGCC | 162 | Caffeoyl-coa o-methyltransferase |
| Cluster-4594.17781 | GGACATACCCAAAGGAGACC | TGGAGCAAGAAGAGACCGT | 165 | P-coumaryl-coa 3'-hydroxylase |
| Cluster-4594.4477 | ACATCCATAGTTCCAGCGG | CCAAGGACCCTGAGAATGA | 124 | Dihydroflavonol 4-reductase |
| Cluster-4594.10931 | AGGGCTACAGGGATTTGAG | TCATTATCGGAGCAACTGG | 104 | Leucoanthocyanidin reductase |
| Cluster-4594.4206 | GTTTCGTCACTTGTCGGTAA | GATTTCCAGATTGCCACAC | 185 | Chalcone isomerase |
| Cluster-4594.6457 | GAAGCAATCATCCAAGGTG | GCAGAGAATCGCATACCAC | 156 | Leucoanthocyanidin reductase |
| Cluster-4594.12512 | GCAACCTTAGCGTCTCTTTC | TCAATGGAGTGGGCAATAG | 160 | Trans-cinnamate 4-monooxygenase |
| Cluster-4594.11055 | AGCATCGTCAAAGAGCCA | TCTCAATCCGTGTCTGTGG | 132 | MYB transcription factor |
| Cluster-4594.13793 | TCATTCCACATAGCCTCCA | TTCTGAGTCGGATGCCAA | 153 | MYB transcription factor |
| Cluster-4594.12981 | TGCTGGTAAGAATCCTGCC | TCTCCATAGGGCTTCCACA | 85 | MYB transcription factor |
| Cluster-4594.7613 | AAAGCACCATCACCATCAG | AGGACTGTGGAAATCGCA | 181 | MYB transcription factor |
| Cluster-4594.1055 | GCCTGGAAGAACAGACAATG | TGTGCTTGGATACATCGCT | 104 | MYB transcription factor |
| Cluster-4594.1156 | GACAATGGCTAACGGTGAC | TTTCAACAACTCCTGCGAC | 197 | MYB transcription factor |
| Cluster-4594.13463 | ACATTGGCTACCTGAGTGG | GGCTTATCCTCCATTACACC | 153 | bHLH transcription factor |
| Cluster-4594.14435 | TTGAGTGTTCTAAGGGCTGA | GTTCTTCCTCTCCAGTCCAC | 126 | bHLH transcription factor |
| Cluster-4594.14756 | TTCTTCATCTTCCTCCTCGT | CCACCCTTATCTTCTTCACC | 120 | bHLH transcription factor |
| Cluster-4594.17259 | CGTGGAGCAGATGAATAAGTC | GCCTAACAAAGCAACAGTCTC | 139 | bHLH transcription factor |
