# Supplementary material for: Integrated Metabolomic and Transcriptomic Analysis Reveals Differential Flavonoid Accumulation and Its Underlying Mechanism in Fruits of Distinct Canarium album Cultivars
Source: Foods. 2022 Aug 21;11(16):2527. doi: 10.3390/foods11162527 (PMC9407539; doi:10.3390/foods11162527)
Supplement: Supplementary file 1 [file foods-11-02527-s001.zip › Table S6. The gene ID numbers and FPKM values of selected MYB and bHLH transcription factors.pdf]

Table S6. The gene ID numbers and FPKM values of selected *MYB* and *bHLH* transcription factors

Gene_ID	T_C1_FPKM	T_C2_FPKM	T_C3_FPKM	T_H1_FPKM	T_H2_FPKM	T_H3_FPKM	Description
Cluster-4594.11055	29.76	38.29	30.79	17.57	17.65	14.30	MYB transcription factor
Cluster-4594.13793	9.00	10.79	10.66	37.12	32.86	29.70	MYB transcription factor
Cluster-4594.12981	8.41	11.07	10.27	3.30	4.40	4.64	MYB transcription factor
Cluster-4594.7613	5.29	5.27	5.56	2.88	1.86	2.39	MYB transcription factor
Cluster-4594.1055	0.74	0.64	1.43	0.96	4.78	4.36	MYB transcription factor
Cluster-4594.1156	1.37	1.48	0.74	7.23	8.15	7.74	MYB transcription factor
Cluster-4594.13463	18.38	22.54	18.42	9.62	10.25	10.11	bHLH transcription factor
Cluster-4594.14435	9.36	11.06	8.83	2.45	2.74	2.12	bHLH transcription factor
Cluster-4594.14756	28.60	38.81	32.21	13.67	10.61	9.27	bHLH transcription factor
Cluster-4594.17259	13.83	14.61	11.24	4.44	5.71	4.62	bHLH transcription factor
