# Supplementary figures and images for: Integrated Metabolomic and Transcriptomic Analysis Reveals Differential Flavonoid Accumulation and Its Underlying Mechanism in Fruits of Distinct Canarium album Cultivars
Source: Foods. 2022 Aug 21;11(16):2527. doi: 10.3390/foods11162527 (PMC9407539; doi:10.3390/foods11162527)

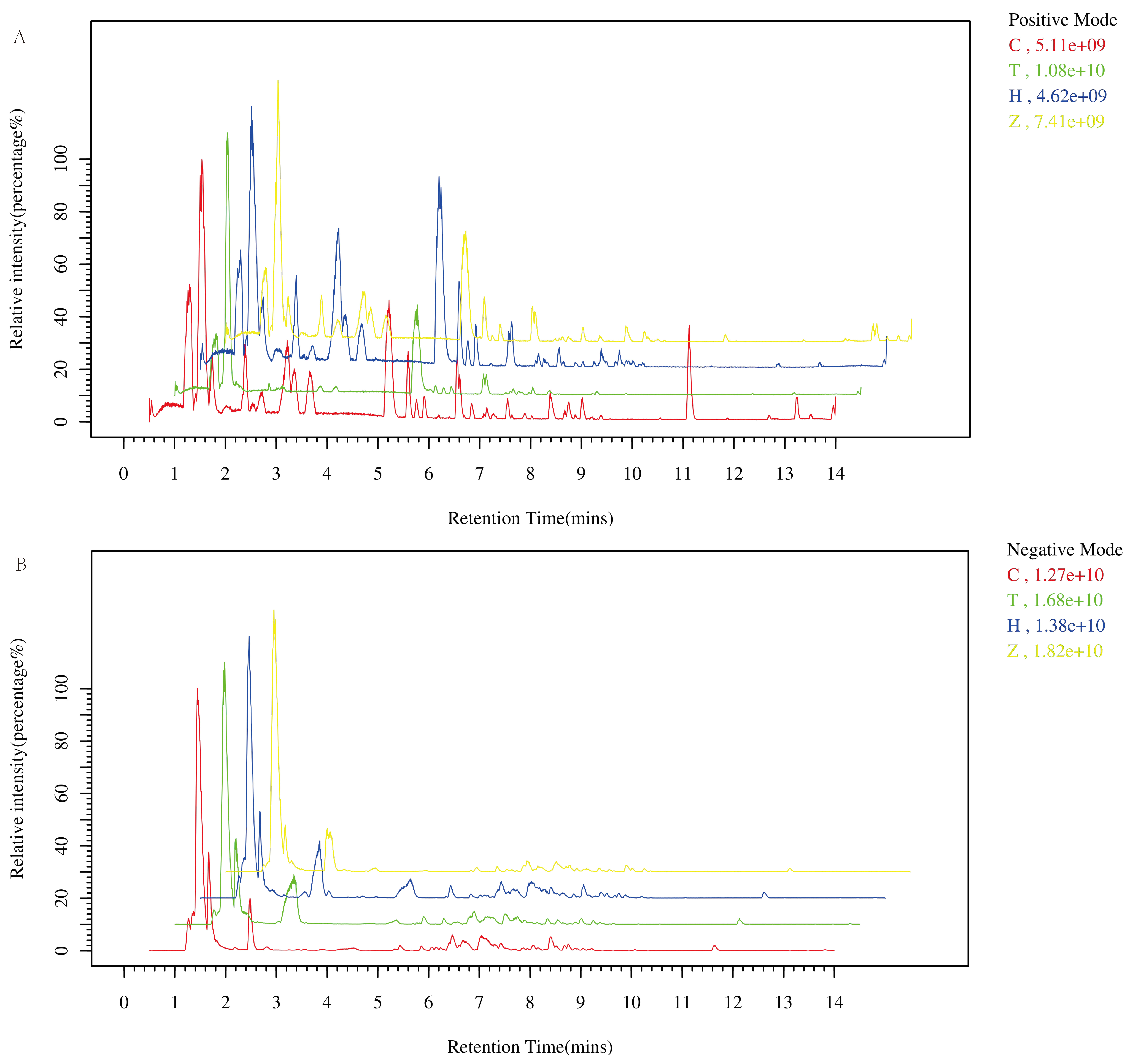

Supplement: Supplementary file 1 [file foods-11-02527-s001.zip › Figure S1. Chromatograms for the LC-MS results of fruits of four Canarium ablum cultivars..jpg]
